# Lipoprotein(a) Gene Polymorphism Increases a Risk Factor for Aortic Valve Calcification

**DOI:** 10.3390/jcdd6030031

**Published:** 2019-08-26

**Authors:** Ugur Ozkan, Fatih Ozcelik, Mustafa Yildiz, Metin Budak

**Affiliations:** 1 Department of Cardiology, Faculty of Medicine, Trakya University, 22030 Edirne, Turkey; 2 Department of Cardiology, Edirne Sultan 1. Murat State Hospital, 22030 Edirne, Turkey; 3 Department of Biophysics, Faculty of Medicine, Trakya University, 22030 Edirne, Turkey; 4 Molecular Research Lab, Prof. Mirko Tos Ear and Hearing Research Center, Trakya University, 22030 Edirne, Turkey

**Keywords:** Lp(a) level, rs10455872, rs3798220, calcific aortic valve stenosis

## Abstract

Calcific aortic valve disease (CAVD) is a multifactorial condition. Both environmental and genetic factors play an important role in its etiology. CAVD exhibits a broad spectrum, varying from mild valve thickening to severe valve calcification and stenosis. Progression of the disease consists of chronic inflammation, lipoprotein deposition, and active leaflet calcification. It is a process similar to coronary artery disease. In this study, we investigated Lp(a) levels and gene polymorphisms associated with calcific aortic stenosis from blood samples after echocardiography in the evaluation of 75 patients diagnosed with CAVD and 77 controls. Blood tests were run in our laboratory to rule out certain risk factors before echocardiography examination. A significant association among smoking, elevated LDL level and creatinine, low albumin levels, Lp(a) level, rs10455872, and rs3798220 polymorphisms may be considered genetic risk factors for the development of calcific aortic stenosis.

## 1. Introduction

Calcific aortic valve disease (CAVD) is a slow progressing comprehensive disorder ranging from mild valve thickening, which is no obstruction to blood flow, to severe calcification with aortic stenosis or impaired leaflet motion. CAVD is the most commonly acquired heart disease in adults, with a prevalence of 2–7% among those over the age of 65 years according to European Cardiology Association (ESC) [1,2,3]. CAVD is predisposed by some risk factors, such as passive degeneration and accumulation of calcific deposits. It is shown to be a dynamic process similar to atherosclerosis [4] and resulting from residual the deposition of lipoproteins, chronic inflammation, and active leaflet calcification [5,6,7].

Early valve disease was characterized by endothelial dysfunction as a prerequisite. Subendothelial accumulation of intracellular lipids and lipoproteins followed by T-cell infiltration and inflammation was observed during the process that began with the deterioration of basement membrane integrity in patients with stenotic occlusion [8,9,10]. Recent studies indicate that genetic variation has been involved in this process, as well as some clinical risk factors, such as age, gender, smoking, diabetes mellitus (DM), hypertension (HT), elevated low-density lipoprotein (LDL) levels and elevated Lp(a) levels. Rashedi et al. showed that the potential effect of a polymorphism in the gene locus associated with lipoprotein(a) synthesis [11,12,13,14].

Lp(a)s are kinds of low-density lipoproteins (LDL) mainly distinguished by their apolipoprotein(a) compounds. Elevated Lp(a) levels are an independent risk factor for chronic heart disease. Lp(a) levels are less associated with CAVD than LDL. Retrospective and cross-sectional studies have shown that Lp(a)s are a risk factor for CAVDs in the early and late stages. The structure of Lp(a) is quite complex. Plasma levels of Lp(a) are controlled or influenced by genetic factors [15,16,17,18,19]. The Lp(a) gene is localized to the q25 region of six chromosomes and encodes the apolipoprotein a component of Lp(a). Changes in this gene may affect plasma levels of Lp(a). Polymorphisms are at the orgins of those changes. Among those polymorphisms, Single nucleotide polymorphisms (*SNPs*) which are defined as rs 10455872 and rs3798220 have been shown to be effective in changing plasma Lp(a) levels [20,21,22].

Although these polymorphisms have been studied in different races around the world, there is no study about the frequency in the Turkish population. In our study; we aimed to shed light on the relationship between Lp(a) levels and genetic polymorphisms factors involved in the development of CAVD in the Turkish population.

## 2. Materials and Methods

### 2.1. Patients

A total of 152 volunteers who met the inclusion criteria were enrolled, including 75 patients and 77 controls. Patients were diagnosed with CAVD by echocardiography (ECHO) while the control group consisted of subjects without CAVD. The volunteers were given explanation of the examination methods. The controls in the study were as follows: Age over 60 years and lack of echocardiographic data for systemic aortic valve disease. Patients in the diagnosed group were over 60 years of age, and had morphological changes presenting with calcification and aortic valve sclerosis, evaluated by ECHO. The ethics of experimentation were approved by the ethics committee of Trakya University. Blood samples were obtained from consenting patients.

Both groups were identified in the echocardiography lab and blood samples (EDTA, 5 cc) were collected. Two genotypes of the Lp(a) gene were identified by means of fluorescence-labeled real-time Polymerase Chain Reaction (Applied Biosystems 7500 Fast Instrument) by utilizing a fluorescent marker (Taqman) after isolating DNA from blood samples. Lp(a) levels were determined by means of ELISA in serum samples.

### 2.2. Echocardiography

Echocardiography in all subjects were performed in the echo laboratory. Measurements were taken after 10 min rest in supine position. Standard transthoracic echocardiography was performed (Vivid 7, GE Vingmed, Horten, Norway).

Parasternal long axis, long-axis projections, apical four-chamber and two-chamber views were obtained. End-diastolic and end-systolic diameters of left atrial and left ventricular walls were measured. Hemodynamic parameters were obtained with continuous-wave Doppler and pulsed-wave. The assessment of severity of valve diseases were categorized as mild, moderate, and severe by using American College of Cardiology/American Heart Association (ACC/AHA) classification. The classification for stenotic lesions and regurgitant was based on the recommendations of the American Society of Echocardiography.

### 2.3. Genotyping

Genomic DNA isolation from peripheral blood was carried out with the manufacturer’s instructions (catalogue number 761001D, Thermo Fisher Scientific, USA). Real-time PCR was performed for rs3798220 and rs10455872 polymorphisms with TaqMan SNP probes by using 2 µl of each DNA sample (catalogue number 4351379, Thermo Fisher Scientific, USA for rs3798220, catalogue number 4351379, Thermo Fisher Scientific, USA for rs10455872).

PCR conditions were as follows: Real-time PCR procedure in Applied Biosystems 7500 Fast Instrument; initiation for 5 minutes at 94 °C, 20 seconds at 94 °C, 20 seconds at 57 °C, 20 seconds at 72 °C, and termination for 10 minutes at 72 °C. Genotyping and melting analysis were performed at the end of each cycle.

### 2.4. ELISA Procedure

Patient serum samples were obtained and stored at −80 °C until use. Quantification of Lp(a) protein (catalogue number LS-F21752, LSBio LifeSpan BioSciences, USA) in serum samples was carried out according to manufacturer’s protocol (PerkinElmer, Waltham, MA). The standard curve was plotted based on the standard and serum Lp(a) level was determined as mg/dL.

### 2.5. Statistical Analysis

Statistical analysis of the study was conducted using SPSS 20 program (IBM Corp, Armonk, NY, USA). Continuous variables were expressed as mean ± standard deviation and qualitative variables as a percentage. Kolmogorov–Smirnov and Shapiro–Wilk tests were utilized to assess normality in the binary comparison of continuous variables, student’s *t*-tests were applied for variables with normal distribution and the Mann–Whitney U test was used for non-normal distribution. Chi-squared (χ2) tests were utilized to compare categorical variables. Genotype frequencies observed in the study was analyzed by means of the HW calculator software and by Pearson’s Chi-squared test for compliance with Hardy–Weinberg distribution, and *p* < 0.05 was accepted as the limit of statistical significance.

## 3. Results

### 3.1. Demographic Characteristics of the Patients

Patient and control groups were compared in terms of demographic characteristics, including age, gender, hypertension (HT), diabetes mellitus (DM), smoking, family history, triglycerides (TG), high-density lipoprotein (HDL), low-density lipoprotein (LDL), and total cholesterol. The control group consisted of 77 people, 41 of them being female and 36 being male. The CAVD group consisted of 75 people; 36 of them were female and 39 were male. There was no statistically significant difference between the two groups with regards to gender (*p* = 0.518). The mean age of control was 71.74 ± 2.17 years, and 72.74 ± 2.36 years was that of the patient group, and no statistically significant difference was found between age and risk factor (*p* = 0.532). There was no statistically significant difference in terms of HT frequency between the patients and the control group (45.45% and 57.33%, respectively) (*p* = 0.143). The difference in DM incidence was also not statistically significant (40.26% in the control group and 46.67% in the CAVD group) (*p* = 0.426). The rate of smoking was 29.87% in the control group and 45.33% in the CAVD group and the difference was statistically significant (*p* = 0.049). LDL measurements were evaluated, and the mean value of LDL was 118.09 ± 4.47 mg/dL in the control group, and 147.97 ± 5.6 mg/dL in the CAVD group; there was a statistically significant difference between the two groups (*p* < 0.001). Evaluation of creatinine levels showed a statistically significant (*p* = 0.002) difference between the control group (1.03 ± 0.09 mg/dL) and the CAVD group (1.29 ± 0.2 mg/dL). Lipoprotein(a) level was 27.05 ± 1.19 mg/dL in the control group and 68.67 ± 1.5 mg/dL in the CAVD group, and there was statistically significant difference (*p* < 0.001) (Table 1).

### 3.2. Allele Frequency

There were 2 SNPs in our original set of loci within genes of potential CAVD. GG genotype was not detected in the CAVD group for rs1055872. A substantial number of SNPs had no allele frequencies for CC and CT of rs3798220, unlike control group. 

Table 2 presents the frequencies of minor allele homozygotes and heterozygotes for all the SNPs genotyped. The AA genotype exhibited a higher frequency in the patient group compared to the control group for rs1055872; however, interestingly GG genotype was not detected in the CAVD group (*p* < 0.001). Regarding other polymorphisms for rs3798220, the entire patient group had the TT genotype, and control group had the CC genotype most prevalently, less so CT and TT genotypes. The rs1055872 polymorphism allele distribution was evaluated; the AA genotype was found in 24.68% of the control group and 97.33% of the CAVD group; the AG genotype was in 14.29% of the control group and 2.67% of the CAVD group; the GG genotype was in 61.04% of the control group and 0% of the CAVD group. The AA genotype exhibited a higher frequency in the patient group compared to the control group; however, the GG genotype was not detected in the CAVD group (*p* < 0.001). The rs3798220 polymorphism allele distribution was evaluated; the CC genotype was in 55.84% of the control group and 0% of the CAVD group; the CT genotype was in 7.79% of the control group and 0% of the CAVD group; the TT genotype was 36.36% of the control group and 100% of the CAVD group. The TT genotype frequency of the patient group was higher than the control group. CC and CT genotypes were not detected in the CAVD group. Statistically, a significant difference was found with high confidence (*p* < 0.001) for CC, CT, and TT (Table 2).

When characteristics of the subgroups (mild, moderate, and severe stenosis) were evaluated, gender, age, HT, smoking, LDL levels, creatinine levels, and Lp(a) levels were similar and there were no statistically significant differences among the subgroups. Only DM was found to be a contributing factor to CAVD progression and had a statistically significant difference (*p* = 0.045) (Table 3). In our study, the level of smoking (*p* = 0.049), LDL (*p* < 0.001), creatinine (*p* < 0.002), albumin (*p* < 0.001), Lp(a) (*p* < 0.001), rs1055872 polymorphism with AA genotype (*p* < 0.001) and rs3798220 polymorphism with TT genotype (*p* < 0.001) were found to be potential risk factors for CAVD development.

The present study included cases referred to our hospital with cardiac symptoms and all of them were underwent echocardiographic evaluation. According to the measurements based on echocardiographic images, subjects were determined as the CAVD group and the control group. The study sample required 75 patients and 77 subjects.

## 4. Discussion

The disease has a progressive pathology. It is characterized by an increase in velocity of 0.3 m/s, a gradient of 7 mmHg, and a decrement in valve area of about 0.1 cm^2^ per year, as indicated in the American Heart Association manual [23]. Since our study was designed as a case-control study, the data for progression could not be evaluated [24,25,26]. Lipoproteins are the most studied risk factors involved in the development and progression of aortic stenosis and mitral valvular stenosis [27]. Lip(a) has a structure that is similar to plasminogen and tissue plasminogen activator and shows competition for the binding site with plasminogen [28,29]. It tends to reduce fibrinolysis due to its structure [30,31]. As it increases plasminogen activator inhibitor-1 secretion, it induces thrombogenicity [32]. Having a cholesterol molecule in its structure increases atherogenesis [31]. Since it carries oxidized phospholipids, which are more atherogenic, it has been shown to be pro-inflammatory [33,34,35,36].

LDL is one of the classic risk factors for atherosclerosis and is accepted as one of the leading factors for the development of CAVD. However, with increasing age, the relationship between LDL-progression of CAVD loosens and becomes less and less in the population over 65 years of age. There was a positive correlation between calcified aortic stenosis and LDL but there was a negative correlation between progressive stenosis and LDL in the subgroup analyses (Table 3). We think that this statistical difference may be the number of subjects in our study. Lp(a) protein level and its gene polymorphisms, which constitute the basis of our study, have recently become popular research. Rashedi et al. emphasized the association between Lp(a) and gene polymorphism for the progression of CAVD. Kamstrup et al. also mentioned the relationship between Lp(a) and CAVD. Thanassoulis et al. investigated the relationship between Lp(a) and rs10455872 polymorphisms in CAVD, and emphasized a significant correlation also [15,16,17]. In our findings, there was a strong positive correlation with the AA genotype of rs10455872 polymorphism and the TT genotype of rs3798220 polymorphism.

As a result of the study using 3145 cases and 3352 control groups among 2100 candidate genes, the European Genome-phenome Archive officially reported that Lp(a) protein causes rs10455872 and rs3798220 alleles for coronary artery disease [37]. Lp(a) contributes to CAVD at different rates depending on ethnicity. The highest prevalence of Lp(a) in CAVD (14.5%) is seen in Caucasians, but the rate of Lp(a) is quite low in Chinese Americans (6.6%) [16,38,39]. rs10455872 (G mutant allele) has been shown to increase the CAVD rate four-fold in the Bulgarian population compared to a control group [16]. The rs3798220(C) polymorphism is a predictor for coronary heart disease in both genders, and it reduces the effect of low dose aspirin therapy in female carriers of the C allele with higher levels of plasma Lp(a) [37,40]. Lp(a) levels varied in different ethnic groups in America for Blacks was 1.51%, for Whites it was 4.27% and for Hispanics it was 42.38%. It seems there is a higher rate in Hispanics [41]. Distributions of these polymorphisms may vary according to populations. In the Danish, AA and TT genotypes of rs10455872 were detected more frequently in the calcific aortic stenosis group, while the GG, CC, and CT genotypes of rs3798220 were found in the control group but not detected in the CAVD group. There was a statistically significant difference between the patients and the control group for all these genotypes. The comparison of genotypes showed a statistically significant difference in CAVD development. Although some studies have investigated the relationship between Lp(a) gene polymorphisms and CAVD development, those studies only focused on polymorphisms without addressing genotypic differences. Moreover, findings of some characteristics were not described according to subgroups of aortic stenosis in some studies. Although clinical and genetic risk factors associated with CAVD were taken into account, the effect of these factors on acceleration progression of CAVD was not investigated [15,16,17].

In our study, the risk factors were investigated by comparing between patient and control groups to determine whether these polymorphisms had any effect on CAVD development. CAVD is often a disease of the elderly population and occurs in 2%–7% of people over 65 years of age. The incidence of symptomatic stenosis increases with age and symptoms of the tricuspid aortic valve start around the age of 70 years [18]. The association between aortic stenosis and classical risk factors of atherosclerosis has been known for several years [19]. The frequency of atherosclerotic coronary artery disease and the incidence of new cardiovascular events are increased in these patients [3,20,21]. LDL level above 125 mg/dL is as a risk factor for the development of aortic stenosis [22]. The mean value of LDL was 118.09 mg/dL in the control group and 147.97 mg/dL in the patient group, and agreed with the aforementioned study [22].

## 5. Conclusions

We discuss our findings in the light of the advantages and limitations of currently available information; such studies are not widely appreciated. While there have been dramatic successes in the identification of a few genetic polymorphisms reported for Lp(a), associations that are complex—environmental, behavioral, and genetic, have been demonstrated to be clinically valid, but few have been shown to have clinical utility. A total of 75 patients with diagnosed CAVD and 77 people without aortic valve anomalies were included in the study. Afterward, patients were divided into a calcific aortic stenosis group and a normal aortic valve group. Demographic and laboratory data were compared for Lp(a) gene polymorphisms. As a result of this comparison, previously identified risk factors, clinical pathologies (age, HT, DM, and male gender) were found to be positively correlated with smoking, LDL, creatinine level, and Lp(a) when compared to the development of CAVD and risk factors for disease development. In addition, there was a statistically significant correlation with the AA genotype of rs1055872 and the TT genotype of the rs3798220 polymorphism (*p* <0.001). The presence of DM was only found to be statistically significant (*p* = 0.049) in the subgroup analysis. A positive correlation was found between low levels of albumin levels and CAVD. None of the patients had a diagnosis of the liver parenchymal disease which could cause liver dysfunction. Therefore, this condition was associated with a negative acute phase reactant of albumin. This has led us to predict that chronic inflammation in the aortic valve may be a component of systemic inflammation, as the process of inflammation in the aortic valve progresses very slowly and spread over the years. Our study also has some limitations that should be taken into consideration. Firstly, these results are dependent on unadjusted estimations, like other genetic and environmental factors. Secondly, this study is considered to have a small number of samples. Therefore, the association of rs10455872 and rs3798220 polymorphisms and CAVD is statistically high, yet this study may be less powerful due to sample size. Despite those limitations, our study is the first evaluation between those polymorphisms and CAVD development in the Turkish population.

## Figures and Tables

**Table 1:** Baseline characteristics of the study population.

Characteristic	Patient Group (*n* = 75)	Control Group (*n* = 77)	*p*-Value
Age	72.74 (70.38–75.11)	71.74 (69.57–73.90)	*0.532*
Women	36 (48%)	41 (53.25%)	*0.518*
Men	39 (52%)	36 (46.75%)	
Diabetes mellitus	35 (46.67%)	31 (40.26%)	*0.426*
Hypertension	43 (57.33)	35 (45.45%)	*0.143*
Smoking	34 (45.33)	23 (29.87)	*0.049*
Low-Density Lipoprotein	147.97 (142.37–153.57)	118.09 (113.62–122.55)	*<0.001*
Creatinine	1.29 (1.09–1.50)	1.03 (0.94–1.12)	*<0.002*
Albumin	3.71 (3.63–3.79)	3.94 (3.87–4.01)	*<0.001*
Lp(a)	68.67 (67.17–70.16)	27.05 (25.86–28.23)	*<0.001*

**Table 2:** Genotype/study population.

Genotype	Patient Group (*n* = 75)	Control Group (*n* = 77)	*p*-Value
rs1055872			*<0.001*
AA	73 (97.33%)	19 (24.68%)	
AG	2 (2.67%)	11 (14.29%)	
GG	0	47 (61.04%)	
Allele			*<0.001*
A	148 (98.67%)	49 (31.80%)	
G	2 (1.33%)	105 (68.2%)	
rs3798220			*<0.001*
CC	0	43 (55.84%)	
CT	0	6 (7.79%)	
TT	75 (100%)	28 (36.36%)	
Allele			*<0.001*
C	0 (0%)	89 (59%)	
T	150(100%)	62 (41%)	

**Table 3:** Baseline characteristics and genotype of patient subgroups.

	Mild Stenosis *n*:16	Moderate Stenosis *n*:28	Severe Stenosis *n*:31	*p*-Value
Male	8(43.75%)	15(46.43%)	13(61.29%)	*0.074*
Female	8(56.25%)	13(53.57%)	18(38.71%)	*0.103*
Age	76.93 (72.58–81.29)	71.32 (67.23–75.41)	71.87 (67.93–74.84)	*0.330*
DM	7(25.00%)	18(46.43%)	10(58.06%)	*0.045*
HT	9(56.25%)	17(53.57%)	17(61.29%)	*0.897*
Smoking	5(43.75%)	14(39.29%)	15(51.61%)	*0.431*
LDL	141.56	150.21	149.25	*0.251*
	(130.39–152.73)	(140.47–159.95)	(139.93–158.5)	
Creatinine	1.13 (0.93–1.33)	1.33 (1.06–1.60)	1.35 (0.92–1.79)	*0.289*
Albumin	3.65 (3.51–3.80)	3.76 (3.60–3.91)	3.70 (3.58–3.81)	*0.363*
Lp(a)	70.28 (68.34–72.23)	69.10 (67.30–70.90)	67.44 (64.26–70.62)	*0.388*
rs1055872				
AA	15(93.75%)	27(96.43%)	31(100%)	
AG	1(6.25%)	1(3.25%)	0	*0.311*
GG	0	0	0	
rs3798220				
CC	0	0	0	
CT	0	0	0	*0.301*
TT	16(100%)	28(100%)	31(100%)	

## References

[1] Freeman R.V., Otto C.M. (2005). Spectrum of calcific aortic valve disease: Pathogenesis, disease progression, and treatment strategies. Circulation.

[2] Iung B., Vahanian A. (2011). Epidemiology of valvular heart disease in the adult. Nat. Rev. Cardiol..

[3] Iung B., Baron G., Butchart E.G., Delahaye F., Gohlke-Barwolf C., Levang O.W., Tornos P., Vanoverschelde J.L., Vermeer F., Boersma E. (2003). A prospective survey of patients with valvular heart disease in Europe: The Euro Heart Survey on Valvular Heart Disease. Eur. Heart J..

[4] Sipahi T., Basak A.A., Ozgen Z., Aksoy A., Omurlu I.K., Palabiyik O., Cakina S., Sener S. (2009). Lack of Evidence for Contribution of Endothelial Nitric Oxide Synthase Intron 4 Vntr Gene Polymorphisms to Development of Ischemic Stroke in Turkish Subjects. Biotechnol. Biotechnol. Equip..

[5] Li C., Xu S., Gotlieb A.I. (2011). The response to valve injury. A paradigm to understand the pathogenesis of heart valve disease. Cardiovasc. Pathol..

[6] Stewart B.F., Siscovick D., Lind B.K., Gardin J.M., Gottdiener J.S., Smith V.E., Kitzman D.W., Otto C.M. (1997). Clinical factors associated with calcific aortic valve disease. Cardiovascular Health Study. J. Am. Coll. Cardiol..

[7] Mohler E.R., Nichols R., Harvey W., Sheridan M., Waller B., Waller B.F. (1991). Development and progression of aortic valve stenosis: Atherosclerosis risk factors—A causal relationship? A clinical morphologic study. Clin. Cardiol..

[8] Novaro G.M., Griffin B.P. (2003). Calcific aortic stenosis: Another face of atherosclerosis?. Clevel. Clin. J. Med..

[9] Otto C.M., Kuusisto J., Reichenbach D.D., Gown A.M., Obrien K.D. (1994). Characterization of the Early Lesion of Degenerative Valvular Aortic-Stenosis - Histological and Immunohistochemical Studies. Circulation.

[10] Helske S., Kupari M., Lindstedt K.A., Kovanen P.T. (2007). Aortic valve stenosis: An active atheroinflammatory process. Curr. Opin. Lipidol..

[11] Wilmshurst P.T., Stevenson R.N., Griffiths H., Lord J.R. (1997). A case-control investigation of the relation between hyperlipidaemia and calcific aortic valve stenosis. Heart.

[12] Cinemre F.B.S., Cinemre H., Yucel A., Degirmencioglu S., Tuten A., Yuksel M.A., Yilmaz N., Gulyasar T., Yildiz M., Bahtiyar N. (2018). The role of selenoprotein P and selenium in the etiopathogenesis of gestational diabetes mellitus: Association with selenoprotein P1 gene (rs3877899) polymorphism. Trace Elem. Electrolytes.

[13] Guldiken B., Sipahi T., Guldiken S., Ustundag S., Budak M., Turgut N., Ozkan H. (2009). Glu298Asp polymorphism of the endothelial nitric oxide synthase gene in Turkish patients with ischemic stroke. Mol. Biol. Rep..

[14] Yücel A., Cinemre F.B., Cinemre H., Yüksel M.A., Tüten A., Yılmaz N., Aydemir B. (2017). Is there a relationship between Selenoprotein P1 (SEPP1) gene polymorphism and gestational diabetes mellitus in Turkish women. J. Hum. Rhythm..

[15] Thanassoulis G., Campbell C.Y., Owens D.S., Smith J.G., Smith A.V., Peloso G.M., Kerr K.F., Pechlivanis S., Budoff M.J., Harris T.B. (2013). Genetic associations with valvular calcification and aortic stenosis. N. Engl. J. Med..

[16] Kamstrup P.R., Tybjaerg-Hansen A., Nordestgaard B.G. (2014). Elevated lipoprotein(a) and risk of aortic valve stenosis in the general population. J. Am. Coll. Cardiol..

[17] Rashedi N., Otto C.M. (2015). Aortic Stenosis: Changing Disease Concepts. J. Cardiovasc. Ultrasound..

[18] Lindman B.R., Clavel M.A., Mathieu P., Iung B., Lancellotti P., Otto C.M., Pibarot P. (2016). Calcific aortic stenosis. Nat. Rev. Dis. Primers..

[19] Rajamannan N.M., Gersh B., Bonow R.O. (2003). Calcific aortic stenosis: From bench to the bedside—Emerging clinical and cellular concepts. Heart.

[20] Mautner G.C., Roberts W.C. (1992). Reported frequency of coronary arterial narrowing by angiogram in patients with valvular aortic stenosis. Am. J. Cardiol..

[21] Aronow W.S., Ahn C., Shirani J., Kronzon I. (1998). Comparison of frequency of new coronary events in older persons with mild, moderate, and severe valvular aortic stenosis with those without aortic stenosis. Am. J. Cardiol..

[22] Aronow W.S., Ahn C., Kronzon I., Goldman M.E. (2001). Association of coronary risk factors and use of statins with progression of mild valvular aortic stenosis in older persons. Am. J. Cardiol..

[23] Anderson J.L., Heidenreich P.A., Barnett P.G., Creager M.A., Fonarow G.C., Gibbons R.J., Halperin J.L., Hlatky M.A., Jacobs A.K., Mark D.B. (2014). ACC/AHA statement on cost/value methodology in clinical practice guidelines and performance measures: A report of the American College of Cardiology/American Heart Association Task Force on Performance Measures and Task Force on Practice Guidelines. J. Am. Coll. Cardiol..

[24] Otto C.M., Burwash I.G., Legget M.E., Munt B.I., Fujioka M., Healy N.L., Kraft C.D., Miyake-Hull C.Y., Schwaegler R.G. (1997). Prospective study of asymptomatic valvular aortic stenosis. Clinical, echocardiographic, and exercise predictors of outcome. Circulation.

[25] Oh J.K., Taliercio C.P., Holmes D.R., Reeder G.S., Bailey K.R., Seward J.B., Tajik A.J. (1988). Prediction of the severity of aortic stenosis by Doppler aortic valve area determination: Prospective Doppler-catheterization correlation in 100 patients. J. Am. Coll. Cardiol..

[26] Pellikka P.A., Sarano M.E., Nishimura R.A., Malouf J.F., Bailey K.R., Scott C.G., Barnes M.E., Tajik A.J. (2005). Outcome of 622 adults with asymptomatic, hemodynamically significant aortic stenosis during prolonged follow-up. Circulation.

[27] Hojo Y., Kumakura H., Kanai H., Iwasaki T., Ichikawa S., Kurabayashi M. (2016). Lipoprotein(a) is a risk factor for aortic and mitral valvular stenosis in peripheral arterial disease. Eur. Heart J. Cardiovasc. Imaging.

[28] Willnow T.E., Goldstein J.L., Orth K., Brown M.S., Herz J. (1992). Low density lipoprotein receptor-related protein and gp330 bind similar ligands, including plasminogen activator-inhibitor complexes and lactoferrin, an inhibitor of chylomicron remnant clearance. J. Biol. Chem..

[29] Anglés-Cano E., Hervio L., Rouy D., Fournier C., Chapman J.M., Laplaud M., Koschinsky M.L. (1994). Effects of lipoprotein (a) on the binding of plasminogen to fibrin and its activation by fibrin-bound tissue-type plasminogen activator. Chem. Phys. Lipids.

[30] Peng S., Xue G., Gong L., Fang C., Chen J., Yuan C., Chen Z., Yao L., Furie B., Huang M. (2017). A long-acting PAI-1 inhibitor reduces thrombus formation. Thromb. Haemost..

[31] Ferretti G., Bacchetti T., Johnston T.P., Banach M., Pirro M., Sahebkar A. (2018). Lipoprotein(a): A missing culprit in the management of athero-thrombosis?. J. Cell. Physiol..

[32] Hosokawa K., Ohnishi-Wada T., Sameshima-Kaneko H., Nagasato T., Miura N., Kikuchi K., Koide T., Maruyama I., Urano T. (2016). Plasminogen activator inhibitor type 1 in platelets induces thrombogenicity by increasing thrombolysis resistance under shear stress in an in-vitro flow chamber model. Thromb. Res..

[33] Yeang C., Wilkinson M.J., Tsimikas S. (2016). Lipoprotein(a) and oxidized phospholipids in calcific aortic valve stenosis. Curr. Opin. Cardiol..

[34] Malaguarnera G., Latteri S., Catalogueania V.E., Malaguarnera M. (2017). Reduction of cardiovascular risk in subjects with high lipoprotein (a) levels. J. Thorac. Dis..

[35] Sreeja S., Manoharan A., Venkataraman K. (2018). Oxidized lipoproteins as the diagnostic target for cardiovascular diseases. Curr. Sci..

[36] Rajamannan N.M., Evans F.J., Aikawa E., Grande-Allen K.J., Demer L.L., Heistad D.D., Simmons C.A., Masters K.S., Mathieu P., O’brien K.D. (2011). Calcific Aortic Valve Disease: Not Simply a Degenerative Process: A Review and Agenda for Research From the National Heart and Lung and Blood Institute Aortic Stenosis Working GroupExecutive Summary: Calcific Aortic Valve Disease–2011 Update. Circulation.

[37] Clarke R., Peden J.F., Hopewell J.C., Kyriakou T., Goel A., Heath S.C., Parish S., Barlera S., Franzosi M.G., Rust S. (2009). Genetic variants associated with Lp (a) lipoprotein level and coronary disease. N. Engl. J. Med..

[38] Cao J., Steffen B.T., Budoff M., Post W.S., Thanassoulis G., Kestenbaum B., McConnell J.P., Warnick R., Guan W., Tsai M.Y. (2016). Lipoprotein (a) levels are associated with subclinical calcific aortic valve disease in white and black individuals: The Multi-Ethnic Study of Atherosclerosis. Arterioscler. Thromb. Vasc. Biol..

[39] Arsenault B.J., Boekholdt S.M., Dubé M.-P., Rhéaume É., Wareham N.J., Khaw K.-T., Sandhu M.S., Tardif J.-C. (2014). Lipoprotein (a) levels, genotype, and incident aortic valve stenosis: A prospective mendelian randomization study and replication in a case–control cohort. Circ. Cardiovasc. Genet..

[40] Chasman D.I., Shiffman D., Zee R.Y., Louie J.Z., Luke M.M., Rowland C.M., Catalogueanese J.J., Buring J.E., Devlin J.J., Ridker P.M. (2009). Polymorphism in the apolipoprotein (a) gene, plasma lipoprotein (a), cardiovascular disease, and low-dose aspirin therapy. Atherosclerosis.

[41] Lee S.-R., Prasad A., Choi Y.-S., Xing C., Clopton P., Witztum J.L., Tsimikas S. (2017). LPA gene, ethnicity, and cardiovascular events. Circulation.

